# The effect of acute non-invasive ventilation on corticospinal pathways to the respiratory muscles in chronic obstructive pulmonary disease

**DOI:** 10.1016/j.resp.2012.05.018

**Published:** 2012-07-31

**Authors:** Nicholas S. Hopkinson, Tarek Sharshar, Mark J. Dayer, Frédéric Lofaso, John Moxham, Michael I. Polkey

**Affiliations:** a NIHR Respiratory Biomedical Research Unit at Royal Brompton and Harefield NHS Foundation Trust and Imperial College London, Royal Brompton Hospital, London SW3 6NP, United Kingdom; b Service de Réanimation Médicale, Hôpital Raymond Poincaré, Garches 92380, France; c Service d’Explorations Fonctionnelles, Hôpital Raymond Poincaré, Garches 92380, France; d Department of Respiratory Medicine, King's College Hospital, Denmark Hill, London SE5 9PJ, United Kingdom

**Keywords:** Transcranial magnetic stimulation, Diaphragm, Motor cortex, Paired stimulation

## Abstract

Chronic obstructive pulmonary disease is associated with altered cortical excitability. The relevance of this to the need for non-invasive ventilation is not known. We assessed the diaphragm response to transcranial magnetic stimulation in terms of motor threshold and latency as well as assessing intracortical excitability using paired stimulation in eight long-term users and six non-users of home ventilation with COPD. Overall, intracortical facilitation was strongly correlated with inspiratory muscle strength (*r*^2^ 0.72, *p* < 0.001) whereas intracortical inhibition was correlated with PaCO_2_ (*r*^2^ 0.51, *p* = 0.01). The two groups did not differ in motor evoked potential or latency, nor in the excitability of intracortical inhibitory or facilitatory circuits assessed using paired stimulation. The acute effect of isocapnic non-invasive ventilation was studied in six established ventilator users. Diaphragm motor evoked potential fell but there was no effect on intracortical facilitation or inhibition, implying an effect of neuromechanical feedback at brainstem or spinal level.

## Highlights

► Patients with COPD who are established on long-term home NIV do not display differences in the excitability of the corticospinal pathways to the respiratory muscles compared to patients with COPD who do not require NIV ► The excitability of intracortical facilitatory and inhibitory circuits assessed using paired transcranial magnetic stimulation is strongly correlated with indices of disease severity in patients with COPD, namely inspiratory muscle strength and hypercapnia respectively. ► Finally, although NIV did reduce the excitability of the corticospinal pathway to the diaphragm it did not, in contrast to previous findings in healthy subjects, alter the excitability of intracortical inhibitory or facilitatory circuits.

## 1 Background

Patients with chronic obstructive pulmonary disease (COPD) have increased neural drive to their respiratory muscles in order to overcome the increased respiratory load that they face (De Troyer et al., 1997; Gandevia et al., 1996; Polkey et al., 1996), but relatively little is known about the cortico-spinal control of the respiratory muscles in COPD. Transcranial magnetic stimulation (TMS) is a technique which allows detailed investigation of corticospinal pathways. A magnetic stimulus applied over the area of the primary motor cortex responsible for the diaphragm elicits an electrical response from the diaphragm, referred to as the motor evoked potential (MEP). Various aspects of the MEP can be measured and may respond to pathophysiological processes (Gandevia and Rothwell, 1987; Gea et al., 1993; Sharshar et al., 2003; Verin et al., 2004). The simplest is the motor threshold which is the lowest intensity of stimulation that elicits a response. The excitability of intracortical inhibitory and facilitatory circuits can be assessed in a non-volitional manner using paired stimulation in which short (<5 ms) interstimulus intervals are inhibitory, whereas at longer (>10 ms) intervals facilitatory pathways are activated with an augmented response. The effects of short and long interval paired-TMS operate via GABA A (the main inhibitory neurotransmitter) and glutaminergic (excitatory neurotransmitter) intracortical circuits respectively (Di Lazzaro et al., 2000; Kujirai et al., 1993; Ziemann et al., 1996a,b).

We have previously demonstrated that in COPD the corticospinal pathway to the diaphragm is more excitable compared to age-matched healthy subjects, with a lower motor threshold and a shorter latency (Hopkinson et al., 2004). Moreover, intracortical facilitation induced by paired-TMS at long interstimulus intervals was markedly attenuated and voluntary efforts beyond 20% of maximal inspiratory pressure did not further facilitate the diaphragm MEP whereas in healthy controls there was a stepwise increase up to 60% of maximum volitional efforts. Taken together these results suggest that the corticospinal pathway to the diaphragm is already highly activated and cannot be further recruited in patients with severe COPD. Given that voluntary activation of the diaphragm appears to be increased in normal subjects at increased lung volumes (McKenzie et al., 1996) and also in patients with COPD compared to controls (Similowski et al., 1991; Topeli et al., 2001), it seems likely that this is an adaptive response to mechanical disadvantage. Consistent with this interpretation the opposite occurs when healthy subjects have their respiratory muscles unloaded by isocapnic non-invasive ventilation (NIV) which leads to an increased diaphragm motor threshold, increased intracortical facilitation and reduced intracortical inhibition (Sharshar et al., 2004b).

The present study addresses three related hypotheses. Firstly, having previously established that there are alterations in cortical excitability in COPD compared to controls (Hopkinson et al., 2004), we hypothesized that these would be related to indices of disease severity or inspiratory muscle impairment. Secondly, we hypothesized that the requirement for long term NIV might be associated with differences in the excitability of intracortical pathways and evaluated this by comparing paired TMS responses in patients who were or were not users of home NIV. Thirdly, we addressed the question of whether the adaptation in the diaphragm motor cortex that occurs in COPD can be reversed by non-invasive ventilation, by comparing responses to single and paired-TMS during spontaneous breathing and isocapnic NIV.

## 2 Methods

### 2.1 Subjects

We studied fourteen male stable outpatients with a diagnosis of COPD consistent with GOLD criteria (Pauwels et al., 2001). The Royal Brompton Hospital Research Ethics Committee approved the study and all subjects provided written, informed consent. Some data from the non-ventilated patients was contained in our previous report (Hopkinson et al., 2004).

All subjects were ex-smokers with a greater than 20 pack year smoking history. Six patients were established on home NIV. When studied, the ventilator users had been on home NIV for a median 33 (range 3–93) months. At the time of their initiation onto NIV the mean PaCO_2_ had been 7.5 (1.2) kPa and PaO_2_ 6.5 (1.3). FEV_1_, TL_CO_ and FRC were 24.8 (4.8), 54 (21) and 149.7 (31)% predicted respectively. The indication for NIV was symptomatic hypercapnia and/or recurrent episodes of Type II respiratory failure. Their lung function and other characteristics *at the time of the study* are described in Table 1 and it should be noted that the ventilator users’ blood gas parameters had improved significantly with treatment. At the time of the study the two patient groups did not differ significantly in their degree of airflow obstruction or lung volumes, but ventilator users had less severe impairment of gas transfer. One ventilated and two unventilated patients declined esophageal catheters so only non-invasive measures were available.

We measured lung volumes, gas transfer (Compact Lab System, Jaeger, Germany) and arterialized capillary blood gas tensions. Esophageal and gastric pressures were measured using catheters passed conventionally connected to differential pressure transducers (Validyne, CA, USA), amplified and displayed online together with transdiaphragmatic pressure (*P*_di_), using LabView software (National Instruments) (Baydur et al., 1982). Maximum sniff nasal pressure (SNiP) was used as a measure of inspiratory muscle strength (Laroche et al., 1988). End-tidal CO_2_ was determined via a nasal catheter connected to a capnograph (PK Morgan Ltd, Gillingham, Kent, UK). Twitch transdiaphragmatic pressure was assessed using bilateral anterolateral magnetic phrenic nerve stimulation as described elsewhere (Mills et al., 1996).

### 2.2 Transcranial magnetic stimulation

The response to TMS was recorded with surface Ag/AgCl electrodes. Electrode position was optimized using supramaximal phrenic nerve stimulation which also provided compound motor action potential (CMAP) amplitude and latency. Signals were acquired into an EMG machine (Synergy, Oxford Instruments, Oxford, UK) with band-pass filtering of signals less than 10 Hz or greater than 10 kHz. To give an assessment of expiratory muscle responses rectus abdominis response was recorded using surface electrodes.

TMS was delivered using Magstim 200 Monopulse units linked via a Bistim timing device (The Magstim Company, Wales) and a 110 mm double cone coil positioned over the vertex (Demoule et al., 2003a; Sharshar et al., 2003). Stimuli were delivered at resting end expiration, assessed from the esophageal and transdiaphragmatic pressure traces, throughout the study and stimuli were repeated if there was evidence of inspiratory activity. An interval of at least 30 s between stimulations was respected.

Motor threshold was defined as the lowest stimulator output producing a MEP of ≥50 μV in ≥5 of 10 trials (Rossini et al., 1994). It was determined by starting at 50% of stimulator output and increasing by 5% increments until threshold was reached.

Paired TMS studies the effect of a conditioning stimulus (CS) of 80% motor threshold on the response to a suprathreshold test stimulus (TS) of 125% threshold, with an interval between them of either 3 or 11 ms to assess inhibitory and facilitatory intracortical circuits, respectively (Demoule et al., 2003b; Hopkinson et al., 2004; Kujirai et al., 1993). Ten paired stimuli were delivered at each interstimulus interval and ten single stimuli at TS intensity in a random order. Values of MEP_3 ms_ and MEP_11 ms_ were expressed as a percentage of MEP_TS_.

The amplitude of the resting MEP_TS_ was normalized in each patient by dividing by the amplitude of the phrenic CMAP obtained during the same study period.

### 2.3 Acute effect of NIV

This was delivered via the patient's own ventilator using a pressure support mode with pressures and back-up rate adjusted to minimize the patient's *P*_di_ curve as far as possible. Subjects were instructed to ‘relax and let the ventilator breathe for you’. PetCO_2_ was kept stable by entraining CO_2_ as required. Once patients had been optimally ventilated for 20 min, diaphragm phrenic nerve CMAP and TMS motor threshold were measured as well as the response to paired stimulation at 3 ms and 11 ms intervals.

Patients sat quietly for 30 min after the end of the ventilation period and a further set of measurements were made.

### 2.4 Data analysis

Data was analyzed using StatView 5.0 software (Abucus Concepts, Berkeley, CA). Variables were compared between groups and between study conditions using Wilcoxon signed rank tests, Mann–Whitney or Chi^2^ tests as appropriate. Univariate linear regression using Pearson correlation coefficient was used to test which disease severity factors were associated with the degree of intracortical facilitation or inhibition. Those with a correlation coefficient of more than 0.3 were included in a forward stepwise regression analysis. Data is given as mean (SD).

## 3 Results

### 3.1 Relation of TMS response to patient characteristics

The diaphragm motor cortex response to transcranial magnetic stimulation during resting breathing did not differ between patients who were (*n* = 8) or were not (*n* = 6) on home NIV in terms of motor threshold, latency or the response to paired stimulation (available in 5 non-ventilated and 6 ventilated patients respectively) with either inhibitory or facilitatory intrastimulus intervals (Table 2). There was also no significant difference in the amplitude of the rectus abdominis response to TMS between the two groups.

**Fig. 1:**
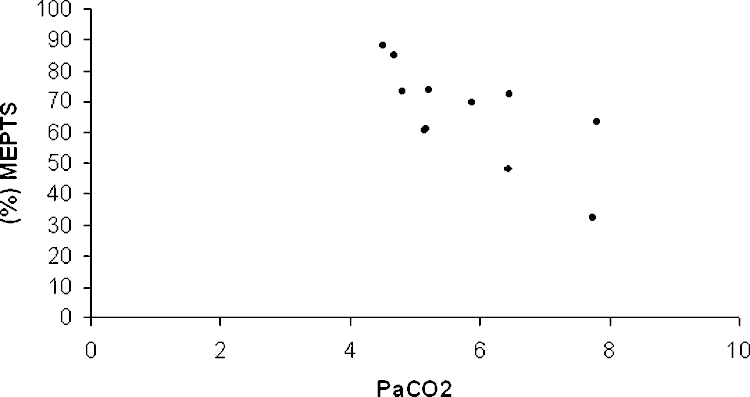
Correlation between intracortical inhibition (reduction in %MEP_TS_ at short interstimulus intervals) and PaCO_2_ (*r*^2^ 0.51, *p* = 0.01).

**Fig. 2:**
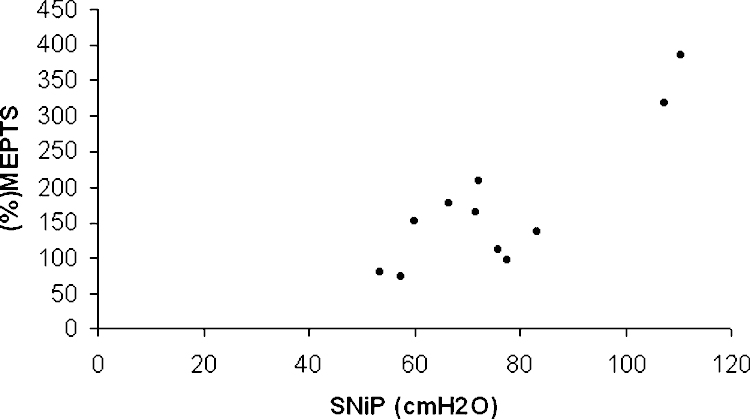
Correlation between intracortical facilitation (increasing %MEP_TS_ at longer interstimulus interval) and inspiratory muscle strength; sniff nasal pressure (SNiP) (*r*^2^ 0.72, *p* = 0.0009).

Correlates of the responses to paired stimulation, assessed in 11 subjects (all six NIV users and five non-users) are given in Table 3. Intracortical inhibition, reflected by the value of normalized MEP_3 ms_, was more pronounced with higher PaCO_2_, lower PaO_2_, lower SNiP, and worse SGRQ. By stepwise analysis only PaCO_2_ was retained as an independent correlate (*r*^2^ 0.51, *p* = 0.01) (Fig. 1). The degree of intracortical facilitation, reflected by the value of normalized MEP_11 ms_, was reduced at higher levels of PaCO_2_ and with increasing airflow obstruction but was not correlated with lung volumes or gas transfer. Using stepwise analysis only SNiP was retained as an independent correlate (*r*^2^ 0.72, *p* = 0.0009) (Table 3 and Fig. 2).

### 3.2 Acute effect of NIV

The acute effect of NIV was studied in six patients who were already established users of nocturnal home NIV. One subject declined to have further stimulations after the end of the period on ventilation so post-NIV data was only available in 5 subjects. NIV significantly reduced the work of breathing with a decrease in diaphragm pressure time product from 269 ± 45 cm H_2_O s^−1^ min^−1^ to 34 ± 13 cm H_2_O s^−1^ min^−1^ (*p* = 0.003). End expiratory pressures at which stimulations were delivered did not differ significantly in the three periods (Table 4). NIV was associated with a significant decrease in normalized amplitude of the diaphragm MEP_TS_ (*p* = 0.02), but it did not alter motor threshold or MEP latency (Table 5). NIV did not alter the excitability of intracortical inhibitory or facilitatory pathways assessed using paired stimulation. NIV was also not associated with significant changes in the amplitude of rectus abdominis MEP_TS_.

## 4 Discussion

The main findings of this study were firstly that the excitability of corticospinal pathways to the respiratory muscles of patients with COPD who have been established on home NIV did not differ from those who do not require NIV. Secondly, the excitability of intracortical facilitatory and inhibitory circuits assessed using paired stimulation was strongly correlated with indices of disease severity, namely inspiratory muscle strength and hypercapnia respectively. Finally, although the acute use of NIV in chronic users did reduce the excitability of the corticospinal pathway to the diaphragm it did not, in contrast to our findings in healthy subjects (Sharshar et al., 2004b), alter the excitability of intracortical inhibitory or facilitatory circuits.

### 4.1 Factors associated with cortical responses in COPD

By studying an expanded cohort of patients we have been able to establish more clearly the relationship between cortical responses and pathophysiological parameters in patients with COPD. Specifically, decreased intracortical facilitation was most closely related to reduced inspiratory muscle strength while greater intracortical inhibition was associated with higher levels of PaCO_2_. This suggests that excitatory circuits are influenced predominantly by neuromechanical feedback and inhibitory ones by chemical inputs. It is interesting in this context to note that isocapnic non-invasive ventilation in healthy subjects had a greater effect on intracortical facilitation than on inhibition supporting a role for neuromechanical feedback as the principle driver for this adaptation (Sharshar et al., 2004b).

The relationship between intracortical facilitation and inspiratory muscle strength implies an adaptation to the failure of the respiratory muscles to generate pressure in COPD since there is a reduction in mechanical output relative to neural output because the respiratory muscles are operating at a mechanical disadvantage. This should not be uncritically accepted as a failure of voluntary drive, firstly because patients with COPD have been shown to be able fully to activate their diaphragm (Topeli et al., 2001) and secondly because we also found a strong correlation between intracortical facilitation and a non-volitional test of diaphragm strength, the TwPdi. Interestingly, although volitional measures of inspiratory muscle strength have tended to improve, at least in patients with restrictive pulmonary disease, following the initiation of NIV, TwPdi does not (Nickol et al., 2005). It is certainly the case that cortical areas, to which vagal afferents including peripheral chemoreceptors and pulmonary stretch receptors project, are involved in the response to respiratory loading and the sensation of breathlessness (Banzett et al., 2000; Isaev et al., 2002).

The neural pathways involved in the control of breathing have the capacity for considerable functional plasticity both in adapting throughout life and in response to stress (Mitchell and Johnson, 2003). In experimental models, hypercapnia is associated in the long term with a depression in phrenic output (Baker et al., 2001). Our finding of a correlation between intracortical inhibition and PaCO_2_ is consistent with this and may represent a novel mechanism involving cortical as well as brainstem responses to explain the phenomenon. It is not clear whether the increased intracortical inhibition observed in COPD patients with increasing hypercapnia is specific for the respiratory muscles or a non-specific response. In favor of a specific process is our previous finding that the corticospinal pathways to the diaphragm and abdominal muscles were more excitable in patients with COPD whereas those to the quadriceps were not, implying that these changes were specific to muscles involved in breathing (Hopkinson et al., 2004). Moreover in another study in healthy subjects, hypercapnia increased diaphragm MEP amplitude and decreased central conduction time but had no effect on the response of a small hand muscle (Straus et al., 2004). In favor of a more generalized process is the fact that a prolonged cortical silent period, a measure of inhibitory tone, has been demonstrated in non-respiratory muscles of a population of patients with obesity hypoventilation and obstructive sleep apnea who were hypercapnic and hypoxic (Civardi et al., 2004). In another study, patients with COPD had reduced intracortical inhibition for the first dorsal interosseous muscle during acute exacerbations which returned to normal when they had been established on long term oxygen therapy and were studied several months later (Oliviero et al., 2002). To our knowledge the effect of hypoxemia and hypercapnia on the diaphragm response to paired TMS has not previously been assessed.

### 4.2 TMS response and long term use of NIV

The factors that determine why some patients with COPD come to need long term NIV remain unclear though lung function, respiratory muscle strength, sleep disturbance, body habitus and respiratory drive may be involved (NAMDRC, 1999). There is evidence from animal models that ventilatory failure is associated with a failure of voluntary motor drive (Ferguson, 1994; Sassoon et al., 1996), and recent human data suggest that maximal central neural output cannot be achieved during exercise either in COPD (Qin et al., 2010) or other pulmonary conditions (Reilly et al., 2011). We hypothesized that the abnormalities in corticospinal transmission that we had previously observed in patients with COPD would be more pronounced in patients who required NIV but this was not confirmed, with no significant difference observed in any TMS parameter assessed. Because the NIV patients had been successfully established on ventilation for several months (and had therefore much improved arterial blood gas parameters) we cannot exclude the possibility that predisposing cortical factors present at the initiation of therapy had been reversed by ventilator use. The issue is further complicated by the fact that nocturnal NIV itself alters daytime blood gas parameters that might themselves alter the response to TMS. Further studies undertaken before and after the initiation of therapy would be required to clarify this.

During the part of the study where the acute effect of NIV was assessed we maintained PetCO_2_ at its baseline value as we wanted to assess the neuromechanical effect of mechanical ventilation alone rather than in combination with any possible chemical effect. This of course differs from conventional ventilator use which by increasing minute ventilation and recruiting alveoli should produce a reduction in PaCO_2_ as well as an increase in PaO_2_.

A related issue is the problem of distinguishing cortical from brainstem and spinal influences on the response to TMS. The observation that the diaphragm response to TMS is the same in normocapnic as in hypocapnic conditions, when the respiratory oscillator is assumed to be silent and also that the response is similar during volitional and hypercapnia driven hyperventilation has been taken as evidence that the corticospinal pathways ‘bypass’ the brainstem (Corfield et al., 1998; Murphy et al., 1990). However, phrenic spinal motor neurons are distinctive in having an ‘automatic’ bulbospinal input as well as a volitional, ‘higher’ corticospinal one, so that their output is dependent both on the amplitude of the corticospinal volley and the output from brainstem respiratory centers. Thus it has been argued that the increase in diaphragm MEP observed during hypercapnia driven hyperventilation is a consequence of an increased brainstem output pre-activating spinal motor neurons rather than an increased cortical response (Straus et al., 2004).

### 4.3 Acute effect of NIV

In a population of healthy controls we have previously found that NIV reduced diaphragm MEP response, increased the excitability of facilitatory circuits and reduced intracortical inhibition, without causing a significant change in rectus abdominis or quadriceps response (Sharshar et al., 2004b). In that study as in the present one NIV did not influence MEP latency. In the current study in COPD patients, although there was a reduction in diaphragm MEP_TS_ during NIV, there was no significant change in the response to paired stimuli. This suggests that the reduction in MEP was principally mediated at a level below the motor cortex. Since isocapnia was maintained this would point to a role for neuromechanical feedback operating either at the spinal level where motor neurons can be preactivated by muscle afferents (Komori et al., 1992) or indirectly via the brainstem respiratory centers which also have afferent input. It has been demonstrated in healthy subjects that inspiratory pressure support ventilation causes hyperventilation since tidal volume rises but respiratory rate does not fall leading to a net fall in CO_2_ (Lofaso et al., 1992). Interestingly hyperventilation with NIV has not been observed during sleep (Morrell et al., 1993) which implies a role for cortical influences. NIV is associated with a reduction in inspiratory activity assessed using diaphragm EMG, which persists even if CO_2_ is corrected (Fauroux et al., 1998), and NIV increases the threshold where a ventilatory response to CO_2_ occurs (Scheid et al., 1994; Simon et al., 1991). Using PET measurements of cerebral blood flow it has been shown that a number of cortical areas are involved in the response to *increases* in inspiratory load (Isaev et al., 2002) (a response which is itself attenuated by sleep) (Santiago et al., 1981), however the diaphragm motor cortex itself was not identified although this may have been at a level below the sensitivity of the test used. Because it is not possible to analyze H-reflex or F-waves for the phrenic nerve it is difficult to assess spinal facilitation directly.

The absence of change in intracortical circuits in response to NIV may represent metaplasticity (Abraham and Bear, 1996), which is a change in the capacity to express plasticity caused by prior exposure; in COPD possibly chronic blood gas derangements or load capacity imbalance in the respiratory muscle pump could be responsible. In the period of spontaneous breathing following NIV, we did not find any change in cortical responses measured compared to baseline.

### 4.4 Critique of the methods

We acknowledge that diaphragm MEP recordings from chest wall electrodes may have been contaminated by signals from either intercostal or abdominal muscles. This was minimized by positioning the surface electrodes close together and optimizing their position in each patient using phrenic nerve stimulation. An alternative would have been to use an esophageal electrode but this would have added significantly to the discomfort of what was already a demanding study for quite severely disabled patients. Furthermore, it has been shown that surface and esophageal electrodes provide the same pattern of response to TMS, either at rest or during voluntary facilitation, in both healthy subjects and patients with COPD (Gea et al., 1993; Sharshar et al., 2005). Moreover, surface electrodes have previously been validated against diaphragm needle EMG (Demoule et al., 2003a) and we were anyway reluctant to use the latter technique because of the risk of pneumothorax during inspiratory effort and in the context of positive pressure ventilation. A related issue is the possibility that changes in the position of the diaphragm relative to the electrodes during NIV could have influenced the response to TMS although the difference between esophageal pressures was not large. TMS responses were therefore normalized to the response to phrenic nerve stimulation to minimize the impact of any peripheral changes.

Ideally we would have performed paired stimulations at a range of interstimulus intervals to produce an interstimulus response curve as described previously (Demoule et al., 2003b; Sharshar et al., 2004a,b). However, this would have considerably increased both the number of stimulations and the duration of the study, so we chose to use only the two interstimulus intervals shown previously to produce the greatest inhibition and facilitation (Hopkinson et al., 2004). Again, to reduce the number of stimulations administered we did not formally assess the motor threshold for the rectus abdominis. However, we have found previously that rectus abdominis threshold in response to stimulation at the vertex is similar to that of the diaphragm both in COPD patients and controls (Hopkinson et al., 2004). A further consideration is that in contrast to the diaphragm, it is not possible to perform peripheral supramaximal stimulation of the abdominal muscles in a manner that is likely to be acceptable to patients (Hopkinson et al., 2010; Suzuki et al., 1999) so it was not possible to normalize the MEP response to allow for any changes in peripheral conduction that might have occurred.

### 4.5 Conclusion

In summary we conclude that a requirement for long-term home NIV in COPD is not associated with changes in the excitability of corticospinal pathways to the respiratory muscles. However we did find, taking the group as a whole, that the facilitatory and inhibitory properties of the intracortical circuits of the diaphragm motor cortex were strongly correlated with inspiratory muscle strength and hypercapnia respectively. While we are cautious in over interpreting the former result we speculate that prolonged exposure to hypercapnia results in greater intracortical inhibition: this could contribute to the pathogenesis of respiratory failure in COPD. Finally, the acute application of NIV did not, in contrast to our previous findings in healthy subjects, alter the facilitatory and inhibitory properties of the diaphragm motor cortex as judged by the response to paired TMS, indicating likely long-term reorganisation of the cortex as a consequence of COPD.

## Conflict of interest

The authors have no conflict of interest.

## Figures and Tables

**Table 1:** Patient demographics, lung function and respiratory muscle strength.

	All patients *n* = 14	Unventilated patients *n* = 8	Ventilated patients *n* = 6
Age	62 (7)	61 (9)	62 (6)
Height (cm)	174 (4)	174 (4)	174 (5)
Weight (kg)	77 (18)	70 (14)	86 (19)
BMI (kg/m^2^)	25 (6)	23 (4)	28 (6)
FEV_1_ (% predicted)	29 (13)	27 (15)	32 (10)
VC (% predicted)	79 (20)	78 (23)	80 (19)
FEV_1_/VC	37 (14)	35 (18)	40 (7)
FRC (% predicted)	216 (62)	240 (63)	184 (46)
TLC (% predicted)	127 (19)	134 (18)	117 (16)
TLco (% predicted)	46 (24)	34.2 (14)	63 (25)†
PaCO_2_ (kPa)	5.7 (1.1)	5.3 (0.7)	6.2 (1.4)
PaO_2_ (kPa)	9.3 (1.3)	9.9 (1.2)	8.4 (0.9)
SNiP (cm H_2_O)	73 (18)	71 (19)	77 (19)
Twitch *P*_di_ (cm H_2_O)^a^	15.6 (5.9)	17.1 (5.9)	13.8 (6.2)
SGRQ	59 (8)	58 (9)	59 (8)

Values are mean (SD). BMI, body mass index; FEV_1_, forced expiratory volume in 1 s; VC, vital capacity; FRC, functional residual capacity; TLC, total lung capacity; TL_CO_, transfer factor; SNiP, sniff nasal pressure; TwPdi, twitch transdiaphragmatic pressure in response to bilateral anterolateral magnetic phrenic nerve stimulation; SGRQ, St George's Respiratory Questionnaire total score. For values in patients pre initiation of NIV see text.

a Available in 11 patients (including 6 unventilated and 5 ventilated patients).

† *p* = 0.03 comparing two patient groups.

**Table 2:** Response to TMS during resting breathing.

	All patients *n* = 14	Unventilated patients *n* = 8	Ventilated patients *n* = 6
Motor threshold (% stimulator output)	58 (14)	53 (13)	63 (14)
Resting MEP_TS_ latency (ms)	15.2 (0.8)	15.1 (0.8)	15.3 (0.8)
Resting CMAP latency (ms)	8.1 (0.2)	8.1 (0.1)	8.0 (0.2)
Resting MEP_TS_ amplitude (%CMAP)	150 (59)	160 (43)	136 (77)
MEP_3 ms_ amplitude (% resting MEP_TS_)	66 (16)	76 (7)^a^	58 (17)
MEP_11 ms_ amplitude (% resting MEP_TS_)	173 (99)	170 (99)^a^	172 (111)
Rectus abdominis MEP_TS_ (μV)	482 (397)	497 (467)	467 (355)

MEP, motor evoked potential in response to transcranial magnetic stimulation; CMAP, compound motor action potential; MEP_TS_, response to test stimulus of 125% resting motor threshold; MEP_3 ms_, MEP_11 ms_, responses to paired stimulation at differing interstimulus intervals. Mann–Whitney test comparing unventilated to ventilated COPD patients all *p* > 0.05.

a Available in only five subjects.

**Table 3:** Correlation between intracortical inhibition (MEP_3 ms_ amplitude) and facilitation (MEP_11 ms_ amplitude) and markers of disease severity.

	Intracortical inhibition (MEP_3 ms_)		Intracortical facilitation (MEP_11 ms_)	
	*R*	*p*	*R*	*p*
PaO_2_ (kPa)	−0.59	0.054	0.37	0.26
PaCO_2_ (kPa)	0.72	0.01*	−0.65	0.03*
FEV_1_ (% predicted)	−0.40	0.22	0.78	0.005*
TLC (% predicted)	−0.54	0.09	−0.02	0.96
FRC (% predicted)	−0.18	0.6	−0.45	0.17
TLco (% predicted)	0.41	0.21	−0.01	0.98
SNiP (cm H_2_O)	−0.61	0.045*	0.85	0.0009*
Twitch *P*_di_ (cm H_2_O)^a^	−0.58	0.13	0.73	0.04*
SGRQ	0.72	0.043*	−0.78	0.022*

PaO_2_, PaCO_2_, arterial partial pressure of oxygen and CO_2_; FEV_1_, forced expiratory volume in 1 s; TLC, total lung capacity; FRC, functional residual capacity; TL_CO_, carbon monoxide transfer factor; SNiP, sniff nasal pressure; Twitch *P*_di_, twitch transdiaphragmatic pressure in response to bilateral anterolateral magnetic phrenic nerve stimulation; SGRQ, St George's Respiratory Questionnaire total score.

a Measured in 11 subjects only.

* *p* < 0.05.

**Table 4:** Values of *P*_ga_, *P*_es_ and *P*_di_ at the time of stimulation before, during and after NIV.

	Before NIV	During NIV	After NIV
*P*_ga_ (cm H_2_O)	15.0 (1.9)	16.1 (2.5)	14.2 (1.6)
*P*_es_ (cm H_2_O)	4.5 (3.1)	5.7 (3.4)	4.6 (1.8)
*P*_di_ (cm H_2_O)	10.5 (2.3)	10.4 (3.8)	9.5 (1.1)

*P*_ga_, gastric pressure; *P*_es_, esophageal pressure; *P*_di_, transdiaphragmatic pressure. Values are mean (SD) for pressures at end expiration when cortical stimulations delivered. All *p* > 0.05.

**Table 5:** Acute effect of NIV on the response to TMS.

	Before NIV *n* = 6	During NIV *n* = 6	After NIV *n* = 5	*p*
Motor threshold (% stimulator output)	63 (14)	66 (18)	71 (18)	0.52
MEP latency (ms)	15.3 (0.8)	14.9 (1.3)	14.8 (1.1)	0.87
Resting MEP_TS_ (% of CMAP)	136 (77)	90 (93)	146 (118)	0.02*
MEP_3 ms_ (% resting MEP_TS_)	58 (17)	76 (41)	79 (28)	0.55
MEP_11 ms_ (% resting MEP_TS_)	172 (111)	232 (146)	188 (71)	0.86
Rectus abdominis MEP_TS_ (μV)	467 (355)	400 (290)	467 (385)	0.87

All data except the bottom row refer to diaphragm responses. MEP, motor evoked potential in response to TMS; CMAP, compound motor action potential response to phrenic nerve stimulation; MEP_TS_, response to stimulus intensity of 125% of motor threshold value; MEP_3 ms_, response to paired stimuli with 3 ms interval; MEP_11 ms_, response to paired stimulation at 11 ms interstimulus interval.

* *p*-Values are for Wilcoxon signed rank test between rest and ventilated values (*p* < 0.05).
